# Educational Setting and SARS-CoV-2 Transmission Among Children With Multisystem Inflammatory Syndrome: A French National Surveillance System

**DOI:** 10.3389/fped.2021.745364

**Published:** 2021-10-26

**Authors:** Celia Guenver, Mehdi Oualha, Corinne Levy, Denise Antona, Fouad Madhi, Julie Toubiana, Noémie Lachaume, Etienne Javouhey, Mathie Lorrot, David Dawei Yang, Michael Levy, Marion Caseris, Caroline Galeotti, Caroline Ovaert, Arnaud Wiedemann, Marie-Laure Girardin, Alexis Rybak, Robert Cohen, Alexandre Belot, François Angoulvant, Naïm Ouldali

**Affiliations:** ^1^Assistance Publique-Hôpitaux de Paris, Department of General Paediatrics, Paediatric Infectious Disease and Internal Medicine, Robert Debré University Hospital, Université de Paris, Paris, France; ^2^Sorbonne Université, Université de Paris, Paris, France; ^3^Assistance Publique-Hôpitaux de Paris, Paediatric Intensive Care Unit, Necker-Enfants Malades University Hospital, Université de Paris, Paris, France; ^4^Centre Hospitalier Intercommunal, Research Centre, Université Paris Est, IMRB-GRC GEMINI, Créteil, France; ^5^ACTIV, Association Clinique et Thérapeutique Infantile du Val-de-Marne, Créteil, France; ^6^Santé Publique France, Agence nationale de Santé publique, Saint-Maurice, France; ^7^Centre Hospitalier Intercommunal, Paediatric Department, Université Paris Est, IMRB-GRC GEMINI, Créteil, France; ^8^Assistance Publique-Hôpitaux de Paris, Department of General Paediatrics and Paediatric Infectious Diseases, Necker-Enfants-Malades University Hospital, Université de Paris, Paris, France; ^9^Institut Pasteur, Biodiversity and Epidemiology of Bacterial Pathogens, Paris, France; ^10^Assistance Publique-Hôpitaux de Paris, Paediatric Emergency Department, Louis Mourier University Hospital, Colombes, France; ^11^Hospices Civils de Lyon, Paediatric Intensive Care Unit, Hopital Femme, Mère Enfant, University of Lyon, Le Born, France; ^12^EA 7426 “Pathophysiology of Injury-Induced Immunosuppression”, University Claude Bernard Lyon 1, Hospices Civils of Lyon, Lyon, France; ^13^Assistance Publique-Hôpitaux de Paris, Department of General Paediatric, Armand Trousseau University Hospital, Sorbonne Université, Paris, France; ^14^Assistance Publique-Hôpitaux de Paris, Paediatric Emergency Department, Necker-Enfants Malades University Hospital, Université de Paris, Paris, France; ^15^Assistance Publique-Hôpitaux de Paris, Paediatric Intensive Care Unit, Robert Debré University Hospital, Université de Paris, Paris, France; ^16^Assistance Publique-Hôpitaux de Paris, Department of Paediatric Rheumatology, Reference Centre for Autoinflammatory Diseases and Amyloidosis (CEREMAIA), Bicêtre University hospital, Université de Paris Saclay, Le Kremlin-Bicêtre, France; ^17^Assistance Publique-Hôpitaux de Marseille, Paediatric and Congenital Cardiology, Timone Hospital Marseille, University Hospital, Marseille, France; ^18^INSERM, Marseille Medical Genetics, UMR 1251, Aix Marseille Université, Marseille, France; ^19^Children's Hospital, University Hospital of Nancy, Paediatric Department, Université de Lorraine, Vandoeuvre les Nancy, France; ^20^INSERM UMRS 1256 NGERE, Nutrition, Genetics, and Environmental Risk Exposure, National Center of Inborn Errors of Metabolism, Université de Lorraine, Vandoeuvre les Nancy, France; ^21^Strasbourg University Hospital, Paediatric Intensive Care Unit, Hautepierre University Hospital, Strasbourg, France; ^22^Assistance Publique-Hôpitaux de Paris, Pediatric Emergency Department, Robert Debré University Hospital, Université de Paris, Paris, France; ^23^Hospices Civils de Lyon, Paediatric Nephrology, Rheumatology, Dermatology, Hopital Femme, Mère Enfant, & Centre International de Recherche en Infectiologie/INSERM U1111, Bron, France; ^24^INSERM, Centre de Recherche des Cordeliers, UMRS 1138, Sorbonne Université, Université de Paris, Paris, France; ^25^Université de Paris, INSERM UMR 1123, ECEVE, Paris, France

**Keywords:** multisystem inflammatory syndrome in children (MIS-C), educational setting, SARS-CoV-2, school, pediatrics-children

## Abstract

**Background:** Multisystem inflammatory syndrome in children (MIS-C) is the most severe form associated with SARS-CoV-2 infection in children. To reduce the spread of SARS-CoV-2 at the population level, educational setting closure have been implemented in many countries. However, the direct benefit of school closure on the MIS-C burden remains to be explored. We aimed to assess the role of educational settings in SARS-CoV-2 transmission among children with MIS-C.

**Methods:** We conducted a French national prospective surveillance of MIS-C, coordinated by Public Health France, from April 2020 to March 2021. During this period, we included all children with MIS-C fulfilling the WHO definition who were reported to Public Health France. For each child, we traced the source of SARS-CoV-2 transmission. The main outcome was the proportion of children with MIS-C, with educational setting-related SARS-CoV-2 infection, during the period of school opening.

**Results:** We included 142 children fulfilling WHO criteria for MIS-C: 104 (70%) cases occurred during school opening periods. In total, 62/104 children (60%, 95%CI [50; 69]) had been contaminated by a household contact and 5/104 in educational settings (5%, 95%CI [2; 11]). Among children with MIS-C occurring during school closure periods, the proportion of household transmission remained similar (66%, 25/38).

**Conclusion:** Children with MIS-C were mainly infected by SARS-CoV-2 within their family environment, and the educational setting played a marginal role in this transmission. This suggests that mitigating school attendance may not reduce substantially the burden of MIS-C.

## Introduction

Multisystem inflammatory syndrome in children (MIS-C) is a life-threatening emerging disease associated with SARS-CoV-2 infection. Cardiovascular complications are common, often requiring inotropic support (1). Thus, MIS-C is by far the most severe form associated with SARS-CoV-2 infection in children and the leading source of burden related to SARS-CoV-2 in this age group (1).

Several non-pharmaceutical interventions implemented during the COVID-19 pandemic included school closure in more than 150 countries (2). After simulation studies (3), several countries including the United Kingdom, Germany and Italy closed educational institutions to mitigate the new wave of COVID-19 in winter 2020-21, and France again in April 2021. This intervention is likely to be discussed again to fight further waves of epidemics.

However, these decisions raised many issues, given their devastating consequences on child well-being and development (4). To help evaluate the public health benefit of these measures over the direct negative consequences, the direct benefit of school closure on the burden of MIS-C, the most severe form of SARS-CoV-2 infection in children, needs to be estimated.

Here we assessed the role of educational setting in SARS-CoV-2 transmission among children with MIS-C.

## Materials and Methods

### Study Design, Patients, and Settings

We used a national prospective surveillance of MIS-C from April 1, 2020 to March 30, 2021. In April 2020, the reporting of all suspected MIS-C cases became mandatory in France, coordinated by the French National Public Health Agency, with a methodology previously published (5, 6). Each suspected MIS-C case was reported by clinicians to the French National Public Health Agency, and was then classified as confirmed MIS-C or not, following World Health Organization (WHO) criteria (6). All children fulfilling WHO criteria for MIS-C up to March 30, 2021 and for whom an electronic case report was completed were included in this study.

### Outcome Measure and Definitions

The primary outcome was the proportion of children with MIS-C with educational setting-related SARS-CoV-2 infection, during the period of school opening.

Secondary outcomes were the proportion of other sources of SARS-CoV-2 infection according to school opening or closure.

In France, schools were closed from March 16 to May 11, 2020, and during summer holidays (from July 1 to August 31, 2020). From the literature, the delay between SARS-CoV-2 infection and MIS-C ranges from 2 to 6 weeks (1, 5, 7). Therefore, we defined the following study periods: “school closure” (from May 1 to September 13, 2020) and “school opening” (rest of the study period). We also conducted a sensitivity analysis considering the following short holiday periods as additional exclusion periods: Autumn break (October 7 to November 1, 2020), Christmas break (December 19, 2020 to January 4, 2021) and February break (February 6–22, 2021).

During the school opening periods of this study, the following non-pharmaceutical interventions were established by the French authorities for educational settings: the use of masks was mandatory for staff and children older than 11, hand washing had to be carried out at a minimum, on arrival in the establishment, before each meal, after going to the toilet, in the evening before returning home. Physical distancing between children in class was recommended (at least one meter) but not compulsory when it was materially impossible. Children and staff had to stay at home when sick or after close contact with someone infected by SARS-CoV-2 (8). Classrooms were ventilated every 3 h for at least 15 min. They were cleaned and disinfected regularly, as are the materials. The restriction of mixing between groups of pupils was not mandatory but each school had to reorganize children's activities to limit the regroupings and crossings between groups.

### SARS-CoV-2 Contact Tracing

The source of SARS-CoV-2 infection, i.e., index case, was defined by a symptomatic contact and/or a confirmed contact (positive SARS-CoV-2 RT-PCR result from a nasopharyngeal swab) during the previous 6 weeks. To define a symptomatic contact, we considered the following symptoms based on the literature (9): cough, ageusia, anosmia, fever, asthenia, influenza-like symptoms, shortness of breath, sore throat, rhinitis, and digestive disorders.

The French National Public Health Agency was involved in contact tracing strategies to identify contacts for children with acute SARS-CoV-2 infection or MIS-C. Furthermore, in France, during school opening periods, recommendations were published to perform SARS-CoV-2 RT-PCR from a nasopharyngeal swab for children with symptoms evoking SARS-COV-2 infection or after contact with cases of SARS-CoV-2 infection (details in Supplementary 1) (10).

### Analysis

We estimated the proportion of the different sources of SARS-CoV-2 infection with their 95% confidence intervals (CIs) by using the adjusted Wald method.

### Ethical Statement

The study was approved by the INSERM Ethics Committee for evaluation (IRB00003888). A written information form validated by the ethics committee was given to all participants. Oral consent was obtained from study participants or parents.

## Results

**Figure 1:**
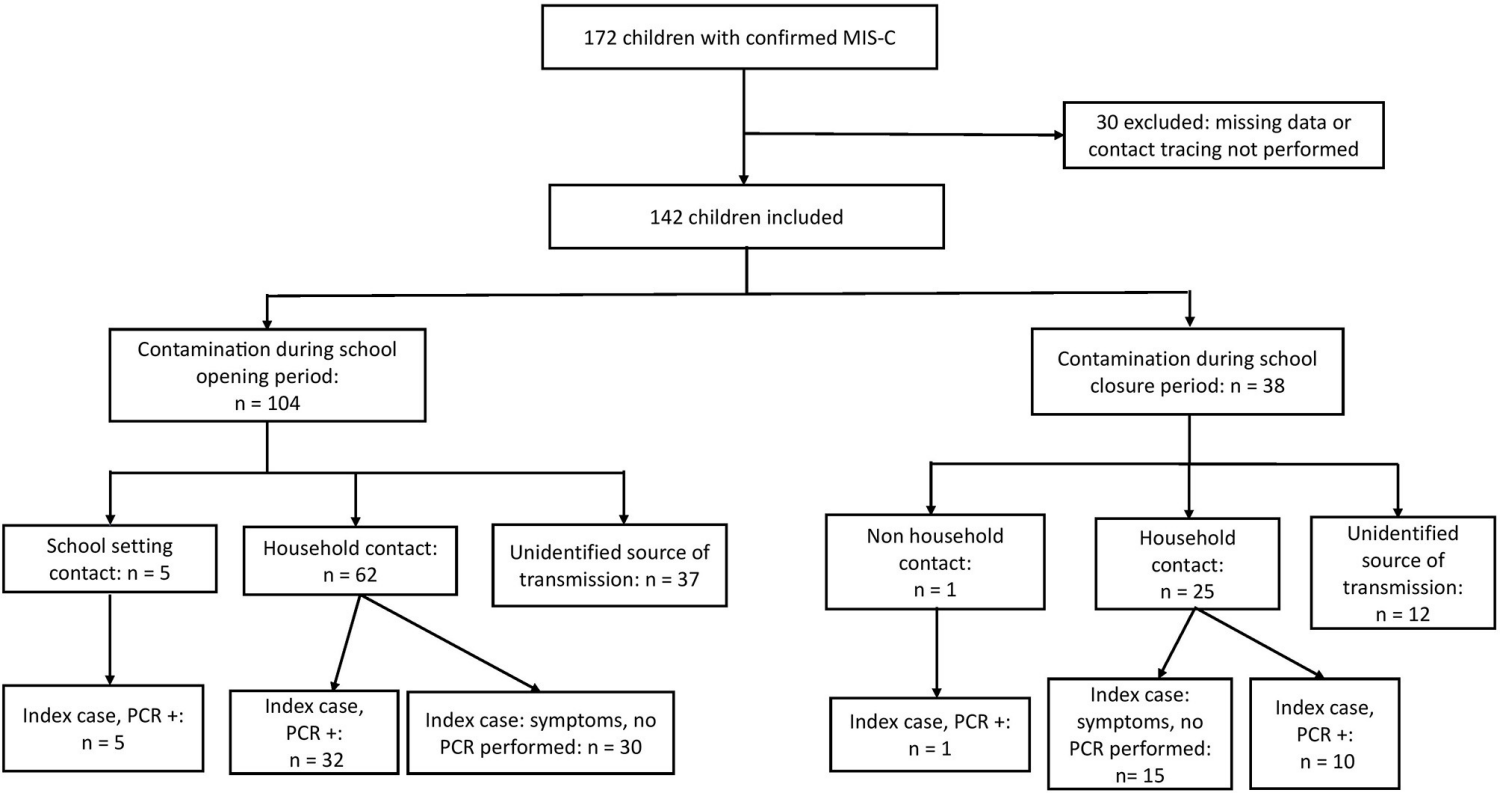
Flow chart of the study participants. MIS-C, multisystem inflammatory syndrome in children.

Among the 172 pediatric patients reported to the French National Public Health Agency with WHO criteria for MIS-C and a completed case report form, 30 children were not included because the source of infection was not investigated or had missing data; 142 children were included (Figure 1).

The median age was 8.3 years (interquartile range [4.8–11.8]) and 62 (43%) were female. The characteristics of patients are in Table 1: 85/142 (60%) had left ventricular dysfunction and 96/142 (68%) were admitted to a pediatric intensive care unit. No deaths were recorded.

**Table 1:** Characteristics of children with multisystem inflammatory syndrome (*N* = 142).

	**Total**	School closure^*^	School opening^*^
	**(*N* = 142)**	**(*N* = 38)**	**(*N* = 104)**
**Clinical characteristics and outcomes**			
Age (years), median (IQR)	8.3 [4.8–11.8]	9.5 [4.0–12.8]	8.1 [5.2–11.4]
Sex ratio (F/M)	0.8	0.7	0.8
Initial left ventricular dysfunction (%)	85 (60)	24 (63)	61 (59)
PICU care (%)	96 (68)	30 (79)	66 (63)
hemodynamic support (%)	62 (44)	17 (45)	45 (43)
**Education setting**			
Kindergarten (%)	45 (32)	14 (37)	31 (30)
Elementary school (%)	52 (36)	8 (21)	44 (42)
Middle school (%)	36 (25)	14 (37)	22 (21)
High school (%)	9 (6)	2 (5)	7 (7)
**Source of SARS-CoV-2 transmission**			
Household contact (%)	87 (61)	25 (66)	62 (60)
Educational setting contact (%)	5 (4)	–^**^	5 (5)
Unidentified source of transmission (%)	49 (35)	12 (32)	37 (35)

*Data are presented as median [Q1–Q3] or number (%). Initial left ventricular dysfunction: Left ventricular ejection fraction <55%*.

* *“School closure” (from May 1 to September 13, 2020); “school opening” (rest of the study period)*.

** *One child was contaminated by a friend, out of the educational setting. IQR, interquartile range; PICU, pediatric intensive care unit*.

For 104/142 (73%) children, SARS-CoV-2 infection occurred during “school opening.” Among them, 62/104 (60%, 95% CI [50; 69]) had been contaminated by a household contact, and 5/104 at school (5%, 95% CI [2; 11]). Among the 62 children infected by household contact, for 32, the index case had a confirmed diagnosis by SARS-CoV-2 RT-PCR, and 30 had symptoms without SARS-CoV-2 RT-PCR performed (Supplementary Table 1). The index case was not identified for 37/104 children (36%, 95% CI [26; 46]).

Among the 38 children for whom the SARS-CoV-2 transmission occurred during “school closure,” 25/38 (66%, 95%CI [49; 80]) had been contaminated by a household contact, and 1/38 (3%, 95% CI [0; 14]) by a friend. The index case was not identified for 12/38 (32%, 95% CI [18; 49]) children.

The sensitivity analysis considering short holiday periods as additional exclusion periods provided similar results [102 children included in the opening school period, of whom 5 (5%) were infected at school, and 60 (59%) by a household contact].

The characteristics of the 5 cases of educational setting-related MIS-C are detailed in Box 1. Four contacts involved classmates; the fifth case was a teacher. All education settings were involved (from kindergarten to high school).

Box 1 **Characteristics of education setting-related SARS-CoV-2 transmission among children with multisystem inflammatory syndrome (MIS-C) (*N* = 5).**

**Table:** 

	**Patient 1**	**Patient 2**	**Patient 3**	**Patient 4**	**Patient 5**
Age, years	15	10	4	12	7
Sex	Male	Male	Female	Male	Male
Educational level	High school	Elementary school	Kindergarten	Middle school	Elementary school
Characteristics of the index case	2 classmates	1 classmate	1 classmate	1 teacher	1 classmate
Delay between contact and MIS-C	4 weeks	4 weeks	4 weeks	4 weeks	2 weeks
Confirmation of SARS-CoV-2 infection for the contact	Yes (NP SARS-CoV-2 RT-PCR)	Yes (NP SARS-CoV-2RT- PCR)	Yes (NP SARS-CoV-2 RT-PCR)	Yes (NP SARS-CoV-2 RT-PCR)	Yes (NP SARS-CoV-2 RT-PCR)
*NP, nasopharyngeal*.					

## Discussion

Children with MIS-C were mainly infected by SARS-CoV-2 within their family environment. Educational settings played a marginal role in the transmission.

Several studies suggested that the circulation of SARS-CoV-2 within educational settings may be low (11–13), when appropriate infection control measures are used (14, 15), as the use of mask, social distancing, and room ventilation (16). This suggestion agrees with many reports showing that children are less infected by SARS-CoV-2 than other age groups (17) and that infected children seem less infectious than adults, thus leading to reduced risk of a transmission chain within schools (11, 12, 18).

Our results could be influenced by an underestimation of SARS-CoV-2 circulation in educational settings. Indeed, symptoms of SARS-CoV-2 infection in children are not specific and are commonly shared by many other respiratory pathogens, which could lead to underdiagnosis of SARS-CoV-2 infection. However, French guidelines recommend performing SARS-CoV-2 RT-PCR with a nasopharyngeal swab for any child attending school with evocative symptoms (10). Furthermore, staff members with any evocative symptoms needed to be tested for SARS-CoV-2 before returning to school, which thus reduced without fully eliminating the risk of underestimating SARS-CoV-2 transmission at school.

The viral inoculum may also play a role in our findings. In numerous common infectious diseases, such as influenza or varicella, the severity of the disease seems linked to the viral inoculum (19). For SARS-CoV-2, the same phenomenon is suspected (20). Because intra-familial contacts are more intense and prolonged than within school, a high viral inoculum may lead to increased risk of severe SARS-CoV-2 infection. Ongoing studies may help elucidate the MIS-C pathophysiology and this specific mechanism.

Several limitations should be discussed. First, the diagnosis of MIS-C relies on non-specific criteria, and misdiagnosis cannot be ruled out. However, all children included in our study fulfilled the WHO criteria for MIS-C. The main clinical characteristics of MIS-C cases included in our study were similar to those in the literature, including median age, rate of cardiac dysfunction, PICU care, and hemodynamic support, enhancing the generalizability of our findings (1). Second, interpreting the minor role of educational setting in SARS-CoV-2 transmission among children with MIS-C should take into account mitigation measures implemented in schools. Third, the source of SARS-CoV-2 infection was not identified in 37/104 cases (36%), which means that patients could have contracted the infection in any setting (family, school, or via social contacts outside home). Several points should be considered. National guidelines to perform SARS-CoV-2 PCR for suspected cases were produced in March 2020. These guidelines regarding SARS-CoV-2 PCR indication did not change thereafter but were gradually applied due to initial lack of availability of PCR testing. The general population was initially not aware about the broad clinical spectrum of SARS-CoV-2 infections, which may have led to under-diagnosis in the first months of the study period. Furthermore, we cannot exclude that transmission from asymptomatic children occurred in educational settings, thus leading to MIS-C with undocumented sources of infection. No systematic test strategies have been implemented in school at a national scale. However, in our study, the proportion of MIS-C with undocumented source of infection remained similar and stable over time in “school closure” and “school opening” periods (32 and 36%), suggesting that educational settings may not play an important role among MIS-C cases with undocumented source of infection.

In conclusion, educational settings seem rarely involved in SARS-CoV-2 transmission among children with MIS-C. Thus, mitigating school attendance does not likely have a direct effect on the burden of MIS-C. By contrast, prolonged school closure have multiple adverse social, educational, health, and economic impacts (21). It might have negative effects on children physical and mental health, with an increase of anxiety and depressive symptoms (22), and could also worsen an already severe food security crisis. Among the consequences in terms of education, there is loss of learning, demotivation, dropping out of school, and increased disparities in academic results, due to unequal access to educational materials. Finally, economic difficulties, an increase in social inequalities and a predicted lower salary for current school children are described in the literature (23). These data have led the French Pediatric Society to publish recommendations for the reopening of schools in September 2020 (10). Likewise, an editorial published in *Science* in September 2020 (24) and an article in the *New England Journal of Medicine* in September 2020 (25) advocate giving maximum priority to the opening of schools with regard to the benefit-risk balance that this measure represents. In this context, our findings may contribute to better weight the expected benefit of school closure against the massive damage they gender, and may be considered by policy-makers when assessing the public health benefit of this intervention.

## Data Availability Statement

The raw data supporting the conclusions of this article will be made available by the authors, without undue reservation.

## Ethics Statement

The studies involving human participants were reviewed and approved by INSERM Ethics Committee for evaluation, Paris, France (IRB00003888). Written informed consent to participate in this study was provided by the participants' legal guardian/next of kin.

## The Following Collaborators Participated in the “French Covid-19 Pediatric Inflammation Consortium”

Investigators: Maelle Selegny, Cinthia Rames (Amiens); Lucas Jeusset, Aurelie Donzeau, Sophie Lety, Bertrand Leboucher (Angers); Agnes Baur (Annecy); Cristian Fedorczuk (Arcachon); Marion Lajus, Philippe Bensaid (Argenteuil); Yacine Laoudi (Aulnay Sous Bois); Charlotte Pons (Avignon); Anne-Cécile Robert, Camille Beaucourt (Besançon); Loïc De Pontual, Camille Aupiais, Alain Lefevre-Utile (Bondy); Muriel Richard, Etienne Goisque, Xavier Iriart, Olivier Brissaud, Pierre Segretin, Julie Molimard, Marion Bailhache (Bordeaux); Elsa Amouyal, Gregoire Benoit, Jean-Emmanuel Kahn, Marie-Clothilde Orcel (Boulognes Billancourt); Lucille Bongiovanni (Brest); Guerder Margaux, Robin Pouyau, Jean-Marie De Guillebon De Resnes, Ellia Mezgueldi, Fleur Cour-Andlauer, Come Horvat, Pierre Poinsot, Cecile Frachette, Antoine Ouziel, Yves Gillet (Bron); Catherine Barrey (Bry Sur Marne); Jacques Brouard, Florence Villedieu, Caroline Faucon, Henri Ginies (Caen); Vathanaksambath Ro, Narcisse Elanga (Cayenne); Vincent Gajdos (Clamart); Romain Basmaci, Nevena Danekova (Colombes); Hadile Mutar (Contamine sur Arve); Sébastien Rouget, Xavier Torterüe (Corbeil Essone); Elodie Nattes, Isabelle Hau, Sandra Biscardi, El Jurdi Houmam, Camille Jung, Ralph Epaud, Céline Delestrain, Adèle Carlier-Gonod (Créteil); Camille Chavy, Benoît Colomb, Stéphanie Litzler-Renault, Denis Semama, Frederic Huet (Dijon); Anne-Marie Zoccarato (Gap); Mayssa Sarakbi (Gonnesse); Guillaume Mortamet, Cécile Bost-Bru, Charlotte Kevorkian-Verguet, Matthias Lachaud (Grenoble); Joachim Bassil (Laval); Caroline Vinit, Véronique Hentgen (Le Chesnay); Pascal Leroux, Valérie Bertrand, Caroline Parrod (Le Havre); Irina Craiu, Isabelle Kone-Paut, Philippe Durand, Pierre Tissiere, Caroline Claude, Guillaume Morelle, Luc Morin, Jordi Miatello, Tamazoust Guiddir, Charlotte Borocco (Le Kremlin-Bicêtre); Frédérique Delion (Les Abymes); Camille Guillot, Stéphane Leteurtre, François Dubos, Morgan Recher, Mylene Jouancastay, Alain Martinot, Valentine Voeusler (Lilles); Jane Languepin (Limoges); Nathalie Garrec, Arnaud Chalvon Demersay (Marne La Vallée); Aurélie Morand, Emmanuelle Bosdure, Noémie Vanel, Fabrice Ughetto, Fabrice Michel (Marseille); Caujolle Marie, Renaud Blonde, Jacqueline Nguyen (Mayotte); Olivier Vignaud, Caroline Masserot-Lureau, François Gouraud, Carine Araujo, Anne Sophie Colas, Claire Ferrua, Anis Larakeb, Sakina Benkaddouss, Laurence Mathivon, Marie Monfort (Meaux); Tara Ingrao (Metz); Sanaa Naji (Mont de Marsans); Mohammed Sehaba (Montargis); Christine Roche (Montbrison); Aurelia Carbasse, Christophe Milesi (Montpellier); Mustapha Mazeghrane (Montreuil); Sandrine Haupt (Mulhouse); Cyril Schweitzer, Nathan Giroux, Noël Boussard (Nancy); Benedicte Romefort, Elise Launay, Christèle Gras-Le Guen, Morgane Dumortier (Nantes); Ahmed Ali, Nathalie Blot (Neuilly Sur Seine); Antoine Tran, Anne Rancurel, Mickael Afanetti, Hervé Haas, Julie Bernardor (Nice); Sophie Odorico (Nîmes); Deborah Talmud, Imen Jhaouat, Françoise Monceaux (Orléans); Anais Chosidow, Anne-Sophie Romain, Emmanuel Grimprel, Pierre-Louis Leger, Jérôme Rambaud, Sandrine Jean, Julie Starck, Yaël Levy, Romain Guedj, Ricardo Carbajal, Pauline Parisot, Caroline Claude, Charlotte Borocco, Marie Pouletty, Olivier Corseri, Camille Ducrocq, Albert Faye, Jean Gaschignard, Isabelle Melki, Ulrich Meinzer, Glory Dingulu, Cherine Benzoïd, Johanna Lokmer, Constance Beyler, Géraldine Poncelet, Richard Wolff, Boris Lacarra, Arielle Maroni, Jérôme Naudin, Guillaume Geslin, Laure Maurice, Anna Deho, Fleur Lebourgeois, Marilyne Chomton, Stephane Dauger, Mathieu Genuini Alexis Rybak, Luigi Titomanlio, Marie Françoise Hurtaux, Guislaine Garcelain, Stéphane Bonacorsi, Philippe Bidet, André Birgy, Sylvain Renolleau, Fabrice Lesage, Florence Moulin, Laurent Dupic, Marion Grimaud, Laure de Saint Blanquat, Claire Heilbronner, Meryl Vedrenne-Cloquet, Elodie Salvador, Matthieu Bendavid, Charles De Marcellus, Judith Chareyre, Joséphine Brisse, Melissa Taylor, Pauline Adnot, Hélène Chappuy, Johanne Auriau, Mathilde Méot, Lucile Houyel, David Drummond, Yael Pinhas, Agathe Debray, Martin Chalumeau, Véronique Abadie, Pierre Frange, Jeremie F Cohen, Slimane Allali, William Curtis, Zahra Belhadjer, Fanny Bajolle, Damien Bonnet, Christophe Delacourt, Brigitte Bader Meunier, Pierre Quartier (Paris); Philippe Blanc, Elisabeth Caron (Poissy); Natacha Maledon (Poitiers); Blandine Robert, Letitia Pantalone, Hanane Kouider (Pontoise); Camille Loeile (Quimper); Clémence Cazau, Gauthier Loron (Reims); Thierry Blanc, Didier Pinquier (Rouen); Simona Gaga (Remiremont); Cécile Vittot, Loubna El Nabhani (Rouen); François Buisson (Saumur); Muriel Prudent (Sens); Hugues Flodrops, Jamal-Bey Karim, Raphaëlle Sarton (St Denis, La Réunion); Fadhila Mokraoui, Simon Escoda, Alexis Mandelcwajg (St Denis); Nina Deschamps (St Malot); Laurent Bonnemains, Sarah-Louisa Mahi, Clara Mertes, Joelle Terzic, Julie Helms, Jeanne Bordet (Strasbourg); Lucas Percheron Ariane Benezech, Thomas Simon, Stephane Decramer, Clement Karsenty, Camille Brehin, (Toulouse); Charlotte Idier (Tours); Soraya Chenichene, Ana-Maria Paulet, Nicoleta Magdolena Ursulescu (Trévenans); Blandine Biot, Céline Manteau, Marie Delattre, Bérengère Dalichoux (Valence); Gladys Beaujour, (Villeneuve Saint Georges).

## Author Contributions

CG, FA, and NO designed the study. CG, NO, MO, FM, NL, and DY collected the data. CG, NO, RC, and FA had a major contribution in the writing of the manuscript. All authors participated in the revision of the manuscript.

## Funding

This study received an unrestricted grant from Pfizer; the French Covid-19 Paediatric Inflammation Consortium received an unrestricted grant from the Square Foundation (Grandir–Fonds de Solidarité Pour L'enfance). The funders had no role in the study design, the collection, analysis, and interpretation of data, the writing of the report; and the decision to submit the manuscript for publication.

## Conflict of Interest

EJ reported receiving grants from CSL Behring. CL reported receiving grants from GlaxoSmithKline, Merck Sharp & Dohme, and Sanofi and personal fees from Pfizer and Merck. RC reported receiving personal fees from GlaxoSmithKline, Pfizer, Sanofi, and Merck Sharp & Dohme. NO report travel grants from GSK, Pfizer and Sanofi, outside the submitted work. The remaining authors declare that the research was conducted in the absence of any commercial or financial relationships that could be construed as a potential conflict of interest.

## Publisher's Note

All claims expressed in this article are solely those of the authors and do not necessarily represent those of their affiliated organizations, or those of the publisher, the editors and the reviewers. Any product that may be evaluated in this article, or claim that may be made by its manufacturer, is not guaranteed or endorsed by the publisher.
