# Supplementary material for: Educational Setting and SARS-CoV-2 Transmission Among Children With Multisystem Inflammatory Syndrome: A French National Surveillance System
Source: Front Pediatr. 2021 Oct 26;9:745364. doi: 10.3389/fped.2021.745364 (PMC8576449; doi:10.3389/fped.2021.745364)
Supplement: Supplementary file 1 [file Data_Sheet_1.docx]

**Supplementary Material**

Supplement S1: French national recommendations for SARS-CoV-2 testing in educational settings.

In France, during school opening periods, recommendations have been published to define the indication of SARS-CoV-2 RT-PCR from a nasopharyngeal swab as follows:^1^

- Any child exposed to a household contact had to be tested before returning to school.
- For children ≥6 years old, SARS-CoV-2 testing was recommended in case of any respiratory or gastrointestinal symptoms when no bacterial infection was identified.
- For children < 6 years old, SARS-CoV-2 testing was recommended in case of severe symptoms, children with a SARS-CoV-2 proven contact; children whose respiratory or gastrointestinal symptoms did not improve within 3 days when no bacterial infection was found.
- Screening of an entire class was warranted if one teacher in the class tested positive for SARS-CoV-2 or if at least two children in the class were symptomatic and tested positive for SARS-CoV-2. There was no systematic screening for asymptomatic forms.

Supplementary Table S1: Symptoms and SARS-COV-2 RT-PCR results from a nasopharyngeal swab of index cases

|  | **Total identified contact cases (N=93)** | **School closure***  **(N=26)** | **School opening ***  **(N=67)** |
| --- | --- | --- | --- |
| **General symptoms** |  |  |  |
| Fever | 20 (22) | 9 (35) | 11 (17) |
| Acute asthenia | 9 (10) | 6 (24) | 3 (5) |
| **Acute upper respiratory tract symptoms** |  |  |  |
| Cough | 16 (17) | 7 (27) | 10 (15) |
| Anosmia ageusia | 16 (17) | 8 (31) | 8 (12) |
| Sore throat | 3 (3) | 0 (0) | 3 (4) |
| Rhinitis | 4 (4) | 1 (4) | 4 (6) |
| Shortness of breath | 3 (3) | 2 (8) | 1 (1) |
| Influenza-like symptoms | 11 (12) | 6 (23) | 5 (7) |
| **Digestive symptoms**** | 4 (4) | 0 (0) | 4 (6) |
| **Positive SARS-COV-2 RT-PCR test with a nasopharyngeal swab** | 48 (52) | 11 (42) | 37 (55) |

Data are n (%). *: “school closure” (from May 1 to September 13, 2020); “school opening” (rest of the study period). **: Digestive symptoms: diarrhea, nausea/vomiting, abdominal pain.

Supplementary table S2A: details about initial left ventricular dysfunction

**Initial left ventricular dysfunction, N = 85/142**

| **Left ventricular ejection fraction** | **20-29%** | **30-39%** | **40-49%** | **50-59%** | **Not quantified** |
| --- | --- | --- | --- | --- | --- |
| **Number of cases (%)** | 3 (3.6) | 19 (22.6) | 35 (41.7) | 19 (22.6) | 9 (10.5) |

Data are n (%).

Supplementary table S2B: details about treatment received (N=142)

|  | **IVIG* alone** | **IgIV + methylprednisolone** | **No specific treatment** | **Methylprednisolone alone** |
| --- | --- | --- | --- | --- |
| **Treatment recieved** | 48 (33.8) | 85 (59.9) | 8 (5.6) | 1(0.7) |

Data are n (%). *IVIg: Intravenous Immunoglobulins

References.

1. R. Cohen,a C. Delacourt,b,⁎ C. Gras-Le Guen,c and E. Launay,c. COVID-19 and schools. Guidelines of the French Pediatric Society. *Archives de pédiatrie*. September 2020.
